# Identification of Two Immune Related Genes Correlated With Aberrant Methylations as Prognosis Signatures for Renal Clear Cell Carcinoma

**DOI:** 10.3389/fgene.2021.750997

**Published:** 2021-12-02

**Authors:** Zhi-Yong Yao, Chaoqung Xing, Yuan-Wu Liu, Xiao-Liang Xing

**Affiliations:** ^1^ School of Public Health and Laboratory Medicine, Hunan University of Medicine, Huaihua, China; ^2^ The First Affiliated Hospital of Hunan University of Medicine, Huaihua, China; ^3^ Beijing Advanced Innovation Center for Food Nutrition and Human Health, China Agricultural University, Beijing, China

**Keywords:** KIRC, DEGs, DMPS, immune-related, prognosis

## Abstract

Almost 75% of renal cancers are renal clear cell carcinomas (KIRC). Accumulative evidence indicates that epigenetic dysregulations are closely related to the development of KIRC. Cancer immunotherapy is an effective treatment for cancers. The aim of this study was to identify immune-related differentially expressed genes (IR-DEGs) associated with aberrant methylations and construct a risk assessment model using these IR-DEGs to predict the prognosis of KIRC. Two IR-DEGs (SLC11A1 and TNFSF14) were identified by differential expression, correlation analysis, and Cox regression analysis, and risk assessment models were established. The area under the receiver operating characteristic (ROC) curve (AUC) was 0.6907. In addition, we found that risk scores were significantly associated with 31 immune cells and factors. Our present study not only shows that two IR-DEGs can be used as prognosis signatures for KIRC, but also provides a strategy for the screening of suitable prognosis signatures associated with aberrant methylation in other cancers.

## Introduction

The incidence and mortality rates of cancer are increasing rapidly worldwide. In 2020, there were about 19.3 million new cancer cases and 10 million cancer deaths (Sung et al., 2021). Renal cancer is one of the most common malignancies, accounting for 2.2% of all new cancer cases (431,288) and 1.8% of all cancer deaths (179,368) (Sung et al., 2021). Renal clear cell carcinoma (KIRC) is the most common subtype, accounting for 75% of all renal cancer cases (Turajlic et al., 2018). So far, KIRC is still difficult to diagnose at the early stage (Alt et al., 2011). Metastases usually appear before the primary tumors are discovered (Alt et al., 2011). Surgical resection is the best treatment for KIRC. However, almost 40% of patients with KIRC who undergo resection will eventually develop distant metastases (Gupta et al., 2008; Porta et al., 2019). Previous studies have also shown that patients with metastatic KIRC have a poor prognosis, with about 10% of patients living for 5 years (Turajlic et al., 2018). Therefore, it is necessary to identify suitable prognosis signatures for patients with KIRC.

Previous studies have shown that renal cancer is believed to arise from cancer stem cells in proximal convoluted tubules, a complex multistep phenomenon involving the accumulation of genetic and epigenetic changes (Prasad et al., 2007; Khan et al., 2019). Epigenetic dysregulations are closely related to the development of renal cancer, such as DNA methylations (Morris and Maher, 2010; Cancer Genome Atlas Research, 2013; Cancer Genome Atlas Research et al., 2016; Morris and Latif, 2017). Nearly 20% of KIRC have a high rate of CpG islands methylations (Cancer Genome Atlas Research, 2013; Hughes et al., 2013; Morris and Latif, 2017). These cancers tissues show high aggressiveness and glycolytic activity (Morris and Latif, 2017). Additionally, previous studies have also shown that several genes are closely related to the cancerogenesis of KIRC and regulated by DNA methylations, such as IDH1/2, *CDO1*, *CTNNB1*, *CDH1*, and *COL1A1* (Ibanez De Caceres et al., 2006; Morris and Maher, 2010; Deckers et al., 2015; Morris and Latif, 2017; Chang et al., 2019)*.*

Cancer immunotherapy is an effective and vital option for cancers patients, such as lung cancer, breast cancer, and pancreatic cancer (Steven et al., 2016; Morrison et al., 2018; Sugie, 2018). Targeted immunotherapy is emerging as a new cornerstone (Deleuze et al., 2020). Cancer immunotherapy can overcome some of the side effects of radiotherapy and chemotherapy. Therefore, it is quite important to identify appropriate signatures to better classify patients and determine the optimal treatment manner and sequence to overcome the drug resistance in patients with KIRC (Deleuze et al., 2020). Therefore, this study aimed to identify immune-related differentially expressed genes associated with aberrant methylations and use them to construct a risk assessment model to predict the prognosis of KIRC.

## Material and Methods

### Data Source and Processing

RNAseq data for 602 samples (72 controls and 530 cancers) and 450K methylations Chip data for 484 samples used in this study were obtained from the cancer genome atlas (TCGA) database. Clinical data for 530 patients with KIRC were downloaded from TCGA database. Identified immune-related genes were downloaded from the ImmPort database (http://www.immport.org). The infiltration data of immune cells and factors were downloaded from Tumor IMmune Estimation Resource (TIMER) (https://cistrome.shinyapps.io/timer/).

DESeq2 in R software (3.6.2) was used to screen the differential expression genes (DEGs) by these criteria: baseMean ≥50, |logFC| ≥ 0.5, adj p-value < 0.05. ChAMP in R software (3.6.2) was used to screen the differential methylations probes (DMPs) by these criteria: |logFC| ≥ 0.3, adj p-value < 0.05. Spearman correlation analysis was used to determine the relationship of immune related DEGs (IR-DEGs) and DMPs by these criteria: R-value ≤ −0.3, p-value < 0.05.

### Survival Analysis

According to the median values, patients with KIRC were divided into a low expression group and a high expression group. Kaplan–Meier (KM) analysis and univariate Cox regression analysis were used to screen the candidate prognosis signatures, followed by least absolute shrinkage and selection operator (LASSO) analysis. Multivariate Cox regression analysis was performed on these IR-DEGs screened by K-M, and univariate Cox regression analysis to obtain the candidate prognostic signatures.

### Risk Assessment Model Construction and Principal Component Analysis

The prognosis signatures determined by multivariate Cox regression analysis were used to construct the risk model. Risk Score = Exp_(SLC11A1)_ *0.4668 + Exp_(TNFSF14)_*0.4458 (Fan et al., 2018; Yao et al., 2020)_._ Principal component analysis (PCA) was used to reduce the dimension and visualize the distribution of patients with KIRC with different risk scores.

### Proteins Interaction and Functional Enrichment Analysis

STRING 11 (https://string-db.org/) and Cytoscape 3.7.2 were used to evaluate and visualize the proteins interactions respectively. DAVID 6.8 was used to carry out the Gene Ontology (GO) and Kyoto Encyclopedia of Genes and Genomes (KEGG) pathway enrichment analysis (https://david.ncifcrf.gov/).

### Statistic Analysis

Unpaired two-tailed Student’s t-test was used to investigate the relationship of the risk scores with the clinical characteristics of KIRC. All results are expressed as mean ± SEM.

## Results

### Identification of IR-DEGs Associated With Aberrant Methylations

**FIGURE 1:**
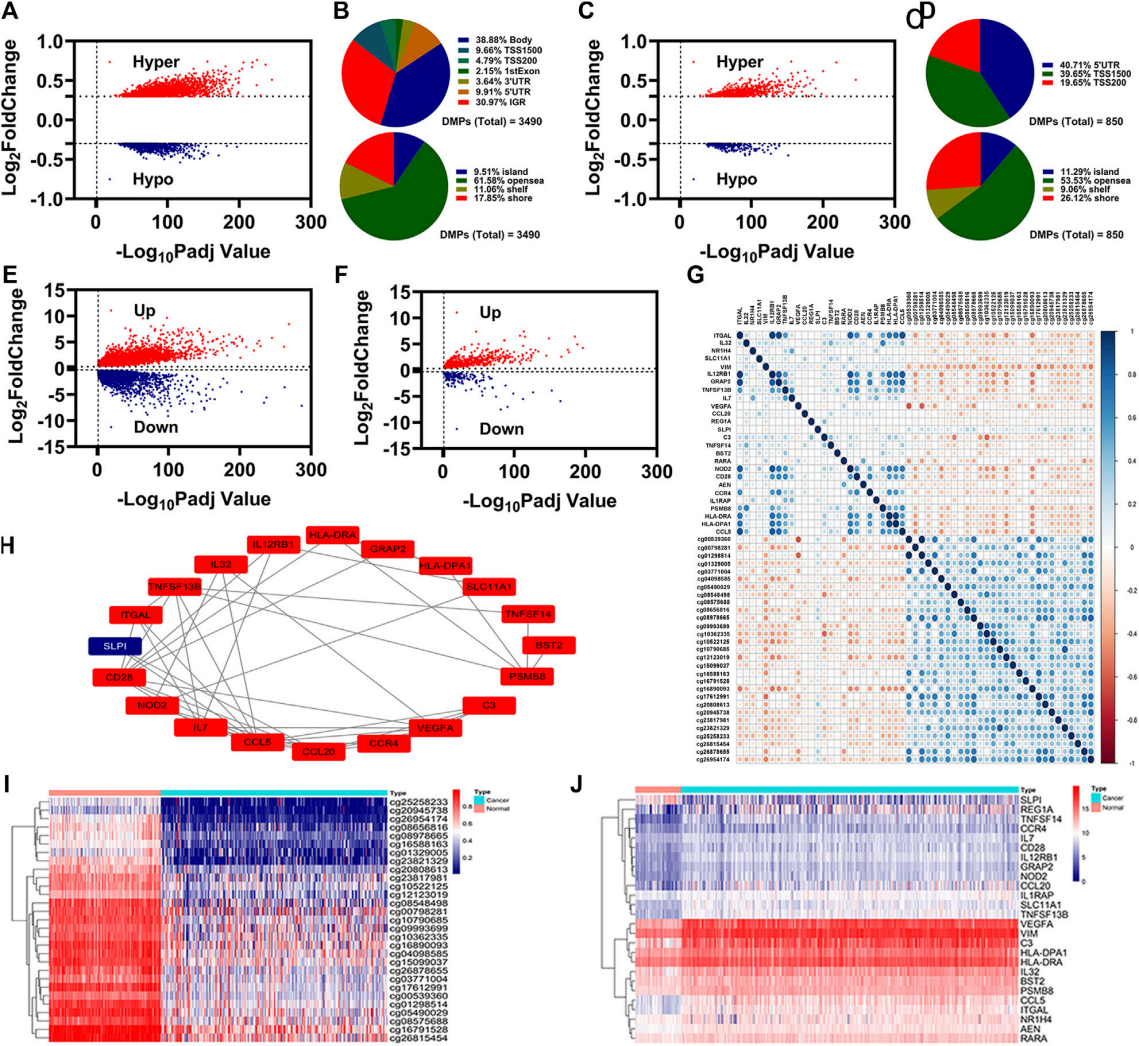
Identification of IR-DEGs associated with aberrant methylations. **(A)**, Volcano plot of DNA methylations status. **(B)**, Distribution of the DNA methylations sites for DMPs. **(C)**, Volcano plot of DNA methylations status for the DMPs in promoter region. **(D)**, Distribution of the DNA methylations sites for the DMPs in promoter region. e-f, Volcano plot of DEGs **(E)** and IR-DEGs **(F)** status. **(G)**, Correlation of 29 DMPs and 26 IR-DEGs. **(H)**, Protein interaction of 26 IR-DEGs. i-j, Heatmap of 29 DMPs **(I)** and 26 IR-DEGs **(J)**.

Through differentially expressed analysis by ChAMP, 3490 DMPs were identified, including 2646 hypermethylation DMPs and 844 hypomethylation DMPs (Figure 1A). Of which, 850 DMPs (618 hypermethylation DMPs and 232 hypomethylation DMPs) were located in the promoter region (5′UTR, TSS200, and TSS1500) (Figure 1C). The distributions of 3490 DMPs and 850 DMPs in the promoter region were displayed in Figures 1B,D, respectively.

Through differentially expressed analysis by DEseq2, 8750 DEGs were identified, including 5319 upregulated DEGs and 3431 downregulated DEGs (Figure 1E). By overlapping with the identified immune-related genes, we obtained 569 upregulated IR-DEGs and 177 downregulated IR-DEGs (Figure 1F).

To know which IR-DEGs were correlated with aberrant methylations, we introduced Spearman correlations analysis for 850 DMPs and 746 IR-DEGs, and found 26 IR-DEGs were negatively correlated with 29 DMPs (Figure 1G). We conducted proteins interaction for these 26 IR-DEGs, and the result was displayed in Figure 1H. The expressions levels of these 26 IR-DEGs and 29 DMPs were displayed in Figures 1I,J.

### Identification of IR-DEGs as Candidate Prognosis Signatures

**FIGURE 2:**
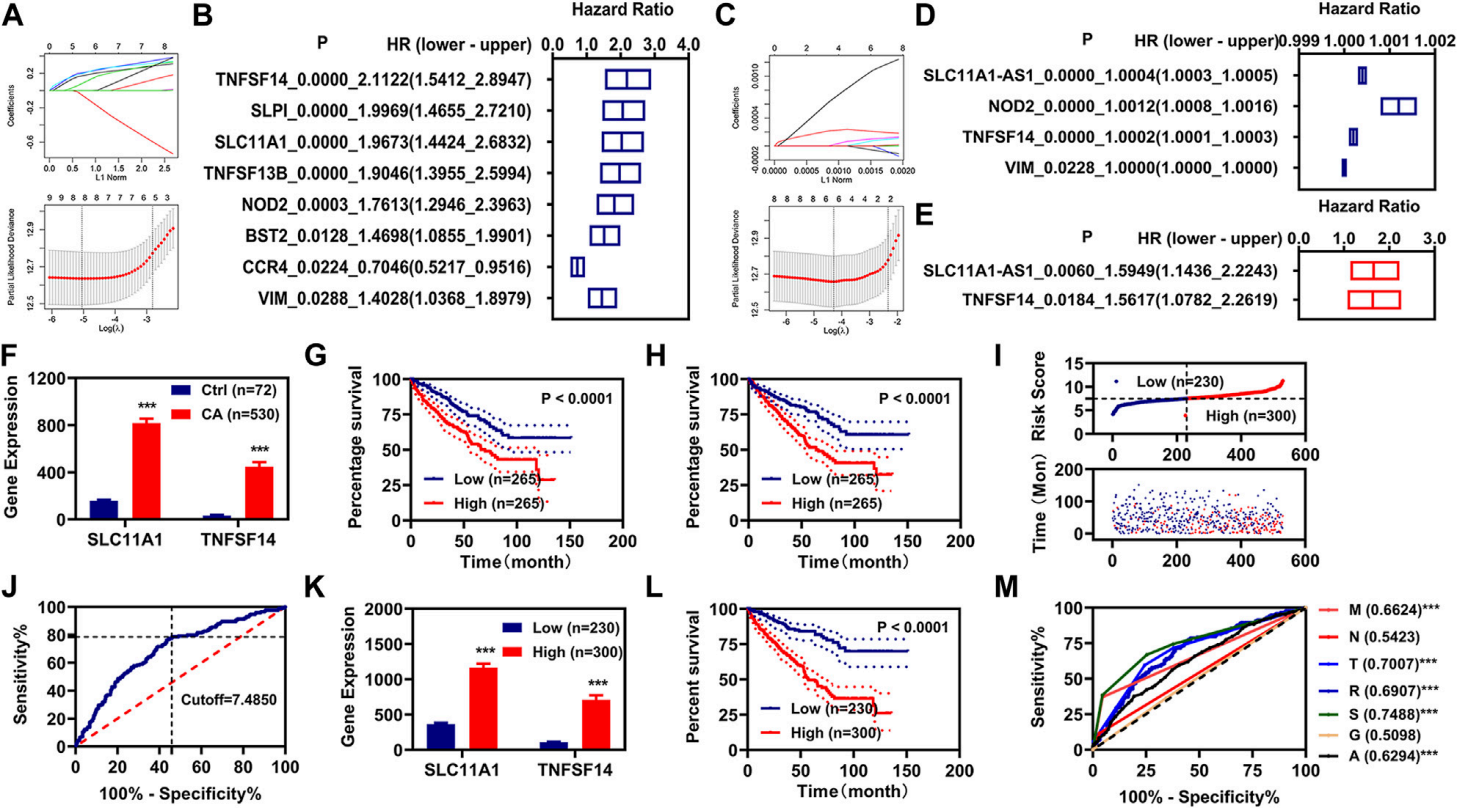
Construction of risk assessment model. **(A,B)**, K-M analysis and LASSO analysis illustrated 8 IR-DEGs. **(C,D)**, Univariate Cox regression analysis and LASSO analysis illustrated 5 IR-DEGs. **(E)**, Multivariate Cox regression illustrated two IR-DEGs (SLC11A1 and TNFSF14). **(F)**, The expression of these two IR-DEGs (SLC11A1 and TNFSF14) in the normal and KIRC cancer patients. **(G,H)**, K-M curve of these two IR-DEGs (SLC11A1 and TNFSF14). **(I)**, Risk scores and survival status for each KIRC. **(J)**, Cutoff value for the risk model. **(K)**, The expression of these two IR-DEGs (SLC11A1 and TNFSF14) in different risk groups. **(L)**, K-M curve of the risk model. **(M)**, ROC curve of different clinical characteristic and the risk model. *p < 0.05, **p < 0.01, ***p < 0.001.

To know the relationships between these 26 IR-DEGs and overall survival (OS) in patients with KIRC, we firstly performed K-M analysis on 26 IR-DEGs followed LASSO analysis, and determined 8 IR-DEGs were associated with the OS in patients with KIRC (Figures 2A,B). We then performed univariate Cox regression analysis on 26 IR-DEGs followed LASSO analysis, and determined 4 IR-DEGs were associated with the OS in patients with KIRC (Figures 2C,D). The overlapping determined IR-DEGs were SLC11A1, VIM, TNFSF14, and NOD2. Subsequently, we performed multivariate Cox regression analysis on these 4 IR-DEGs, and found SLC11A1 and TNFSF14 were associated with the OS in patients with KIRC independently (Figure 2E). The expressions of these two IR-DEGs were significantly increased in patients with KIRC (Figure 2F). Patients with high expressions of SLC11A1 or TNFSF14 had poor OS (Figures 2G,H).

### Construction of Risk Assessment Model

We constructed a risk assessment model using SLC11A1 and TNFSF14. The risk score and survival status of each KIRC patient were displayed in Figure 2I. We used the optimal cutoff value to regroup the patients with KIRC into low-risk and high-risk groups (Figure 2J). The expressions of these two IR-DEGs were also significantly increased in the patients with KIRC with high-risk scores (Figure 2K). Patients with KIRC with high-risk scores had poor OS (Figure 2L). Then the ROC curve was plotted and the AUC value was calculated, as shown in Figure 2M.

### Correlation Analysis of Risk Scores With Clinical Characteristics

**FIGURE 3:**
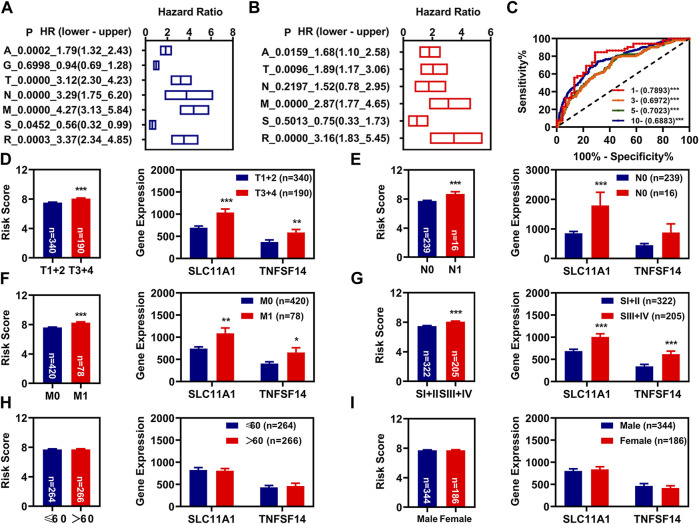
Independent prognosis factors and correlation analysis. **(A)**, K-M analysis of prognosis factors. **(B)**, Multivariate Cox regression analysis of prognosis factors. **(C)**, The 1-year, 3-year, 5-year, and 10-year ROC of the risk model show that all AUC values were over 0.60. **(D–G)**, Correlation of risk values **(left)** and these two FR-DELs **(right)** expressions with the pathologic T **(D)**, pathologic N **(E)**, pathologic N **(F)**, pathologic stage **(G)**, age **(H)**, and gender **(I)**. *p < 0.05, **p < 0.01, ***p < 0.001.

We performed the K-M and multivariate Cox regression analysis on the clinical characteristics and risk models of patients with KIRC, and found that age, pathologic TNM, pathologic stage, and risk model were correlated with the OS of patients with KIRC, as measured by K-M analysis (Figure 3A). Age, pathologic TM, and risk model were correlated with the OS of patients with KIRC independently, as measured by multivariate Cox regression analysis (Figure 3B). By retrospective examination, we found that the AUC values of risk models were comparable to pathologic T and slightly higher than that of pathologic M and age (Figure 2M). The AUC value of the risk model at 1, 3, 5, and 10 years was over 0.60 (Figure 3C).

Subsequently, we also investigated the relationship between the risk scores and different clinical characteristics. The results suggested that the risk scores of patients with KIRC with pathological stage T3+4, N1, M1, III + IV patients were higher than these of patients with KIRC with pathological stage T1+2, N0, M0, I + II patients, and the risk scores of patients with KIRC with different age and sex were comparable (Figures 3D–I left). SLC11A1 was significantly increased in patients with KIRC with pathologic T3+4, N1, M1, and III + IV. TNFSF14 was significantly increased in patients with KIRC with pathologic T3+4, M1, and III + IV. There was no significant difference for TNFSF14 in different pathologic N (Figures 3D–I right).

### PCA and Functional Enrichment Analysis

**FIGURE 4:**
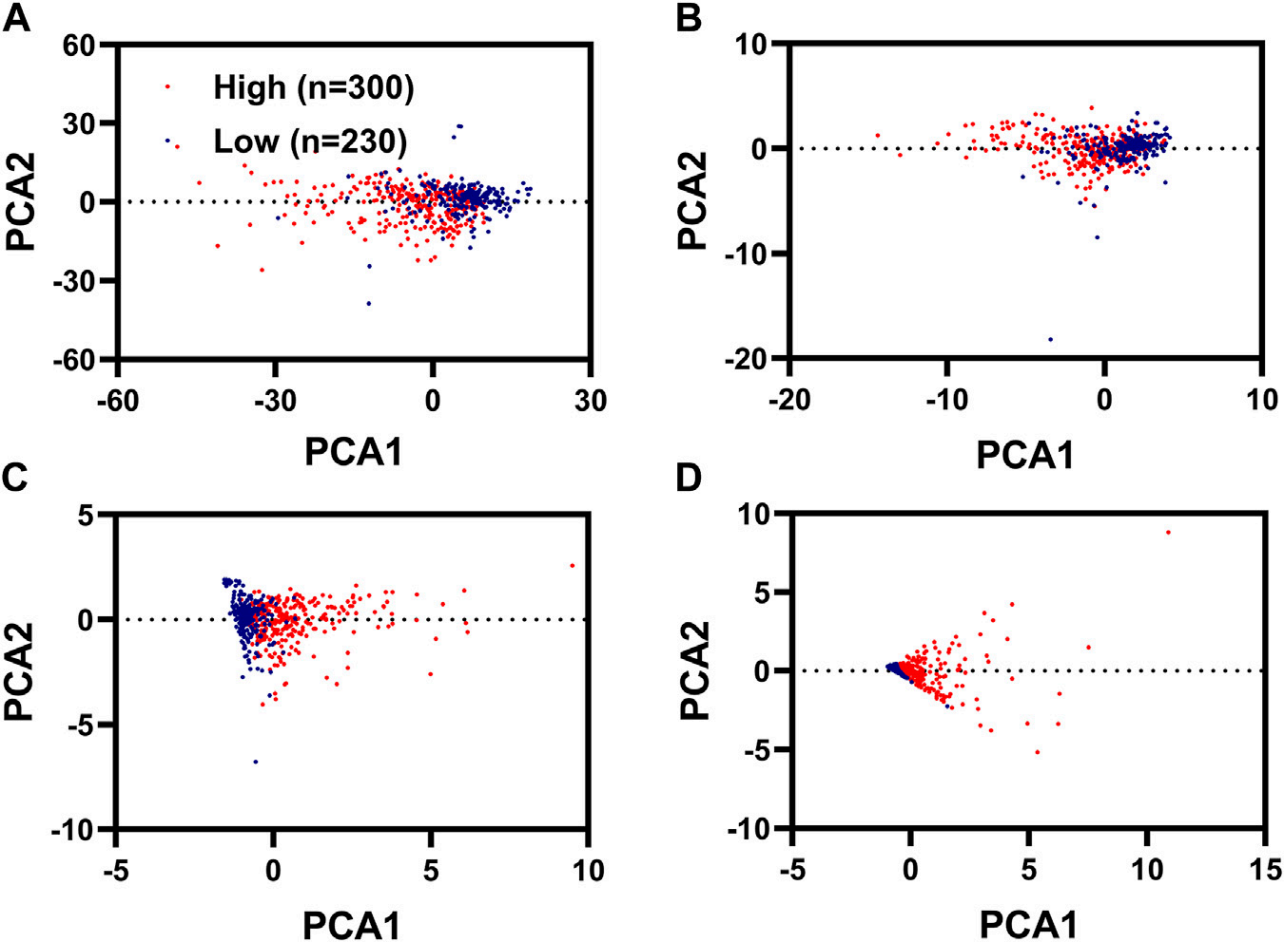
PCA analysis for KIRC with different risk scores. PCA plots displayed the distribution of patients with renal cancer with high risk scores and low risk scores based on 746 FR-DEGs filterer by differentially expressed analysis **(A)**, 26 FR-DEGs filtered by Spearman correlation analysis **(B)**, 4 FR-DEGs filtered by K-M analysis and univariate Cox regression analysis **(C)**, two FR-DEGs filtered by multivariate Cox regression analysis **(D)**.

PCA analysis was used to reduce the dimension and visualize the distribution of patients with KIRC with different risk scores. We could well distinguish patients with KIRC with high-risk scores from the patients with KIRC with low-risk scores using these four IR-DEGs (SLC11A1, VIM, TNFSF14, and NOD2) filtered by KM analysis and univariate Cox regression analysis (Figure 4C) and these two IR-DEGs (SLC11A1 and TNFSF14) filtered by multivariate Cox regression analysis (Figure 4D). We could not use these 746 ID-EGs filtered by differentially expressed analysis (Figure 4A) and these 26 IR-DEGs filtered by Spearman correlation to distinguish between high-risk and low-risk patients (Figure 4B).

**FIGURE 5:**
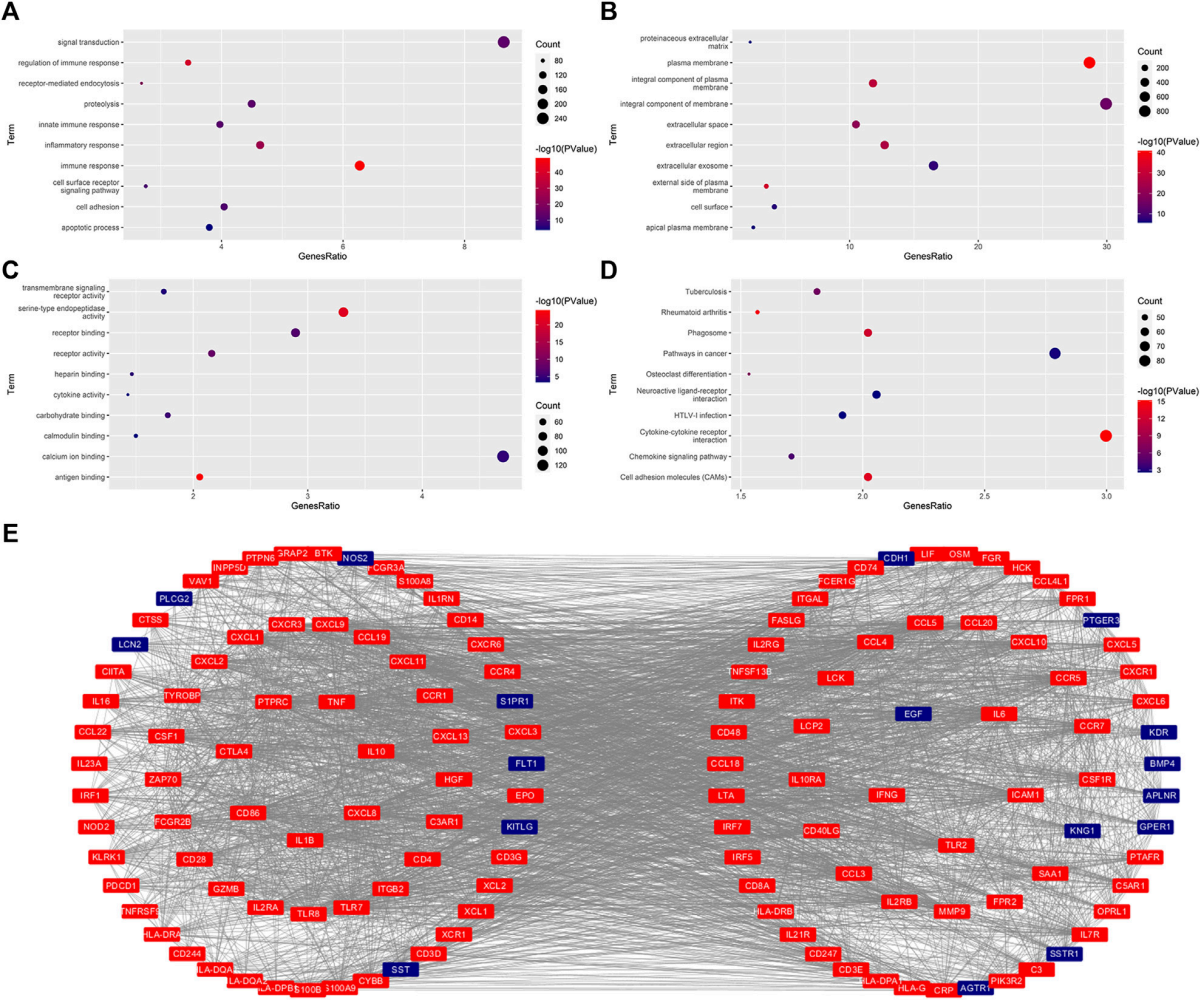
Functional enrichment analysis and protein interaction analysis. **(A–C)**, The significantly enriched GO term (top 10). BP, Biological Process **(A)**. CC, Cellular Component **(B)**. MF, Molecular Functions **(C)**. **(D)**, The significantly enriched KEGG pathway (top 10). **(E)**, The protein interaction for these FR-DEGs with their degree ≥average (32.1).

We then re-performed the differential expression analysis for these patients with KIRC with different risk scores, and identified 3333 DEGs (2220 upregulated DEGs and 1113 downregulated DEGs) (Supplementary Figure S1). GO analysis indicated that there were 73 BP, 24 CC, and 20 MF that were enriched significantly with p value <0.05 and FRD <0.05 (Supplementary Table S1). The BB, CC, and MF with the number of genes ranked in the top 10 are shown in Figures 5A–C. KEGG analysis indicated that 41 signaling pathways were enriched with p value <0.05 and FRD <0.05 (Supplementary Table S2). The signaling pathways with the number of genes ranked in the top 10 are shown in Figure 5D. Of these 3333 DEGs, 436 were immune-related DEGs. We also performed the proteins interaction analysis for these 436 IR-DEGs. In general, the more a gene interacts with other genes, the more important its function is. We obtained 136 IR-DEGs, which were higher than the average (32.1). The interactions of these 136 IR-DEGs were shown in Figure 5E.

### Correlation Analysis of Risk Scores With Immune Infiltration

**FIGURE 6:**
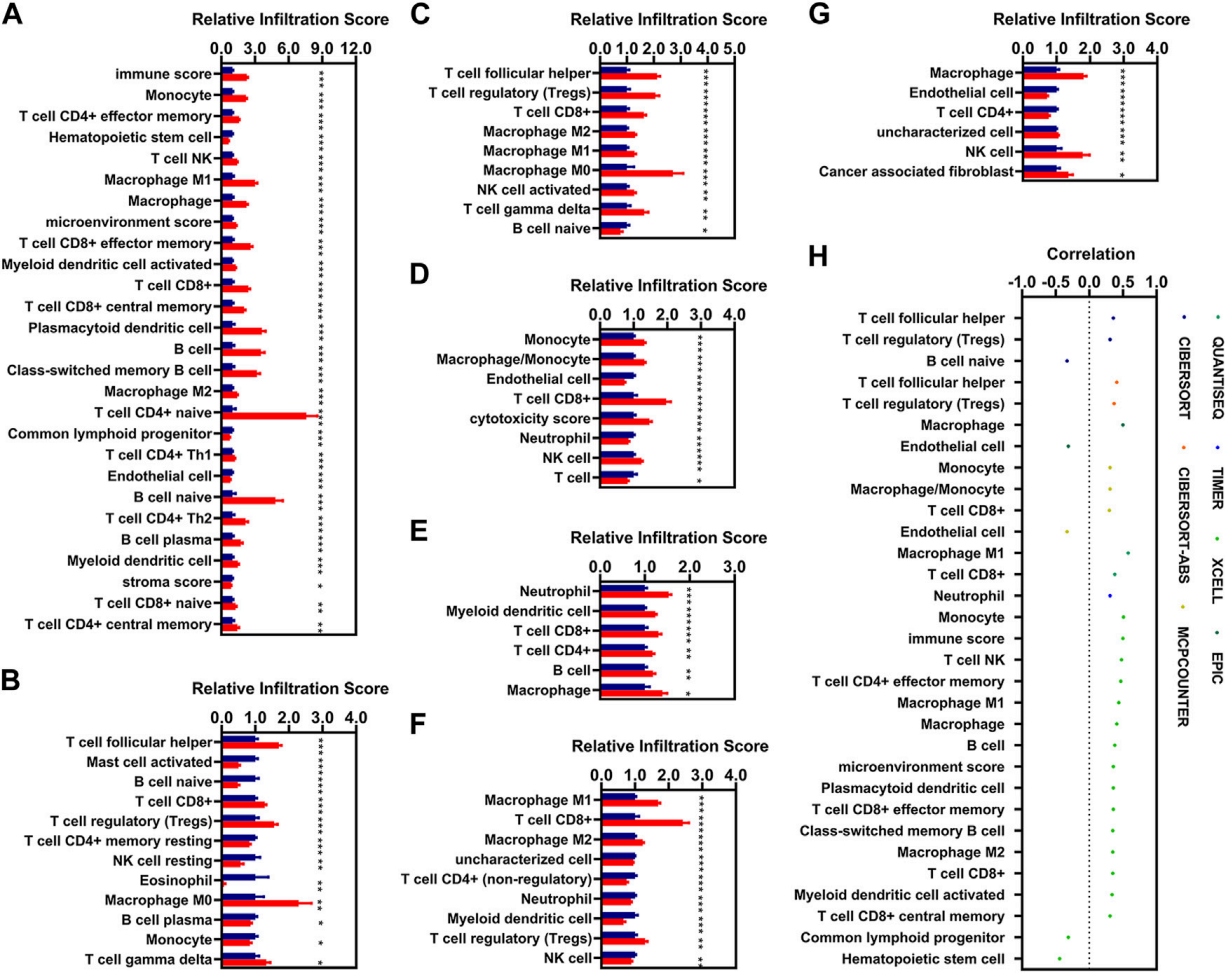
Correlations analysis of risk scores with immune infiltration. **(A–G)**, The expression of the immune cells and factors with the risk model [**(A)**, XCELL. **(B)**, CIBERSORT. **(C)**, CIBERSORT-ABS. **(D)**, MCPCOUNTER. **(E)**, TIMER. **(F)**, QUANTISEQ. **(G)**, EPIC). **(H)**, Correlation of the risk models with 31 immune cells and factors (|R| > 0.3, *p* < 0.05). * means *p* < 0.05, ** means *p* < 0.01, *** means *p* < 0.001.

In the present study, we aimed to identify IR-DEGs associated with aberrant methylations as prognosis signatures. We firstly investigated the relationships of immune cell infiltration with KIRC, and found 67 and 21 immune cells and factors were significantly increased and decreased in patients with KIRC respectively (Supplementary Table S3). Of these, there were 77 different immune cells and factors that were significantly different between low-risk and high-risk patients (Figures 6A–G). We then introduced Spearman correlation analysis for the risk model with these 77 immune cells and factors, and found that 26 and 5 immune cells and factors were positively and negatively correlated with the risk scores respectively (Figure 6H).

## Discussions

Renal cancer is one of the most common malignancies. Accumulative studies indicated that aberrant DNA methylations are involved in the development of cancers (Morris and Maher, 2010; Cancer Genome Atlas Research, 2013; Cancer Genome Atlas Research et al., 2016; Morris and Latif, 2017). Radiotherapy and chemotherapy are common strategies for cancers accompanied by surgery. Cancer immunotherapy is a new alternative option for cancers that could overcome the nonspecific problems of radiotherapy and chemotherapy. It is fairly important to identify IR-DEGs as prognosis signatures to predict the outcome for KIRC. In the present study, we found that two IR-DEGs (SLC11A1 and TNFSF14) were significantly increased in the patients with KIRC and patients with KIRC with high-risk scores. High expression of these two IR-DEGs (SLC11A1 and TNFSF14) displayed worse OS. These two IR-DEGs could used to be prognosis signatures for KIRC.

SLC11A1 is a member of the solute carrier family 11 (proton-coupled divalent metal ion transporters) family. It is associated with susceptibility to various autoimmune and infectious diseases. However, several studies have demonstrated that SLC11A1 is also closely related to cancers. Zaahl et al. (2005) found that genetic variations in both the promoter region and intron 1 of the SLC11A1 were associated with esophageal cancer. Takashima et al. (2018) found that glioblastoma multiforme (GBM) patients with high expression of SLC11A1 displayed worse OS. SLC11A1 could be a promising predictor of the prognoses of GBM patients and used to develop effective GBM treatment strategies (Takashima et al., 2018). The results of our present study were consistent with previous results, and further suggested that SLC11A1 was closely related to cancers and may be used as their prognosis biomarker.

TNFSF14 (TNF superfamily member 14) is a member of the tumor necrosis factor (TNF) ligand family, encodes by *TNFSF14*. The expression of TNFSF14 within tumors has profound effects on host immune responses against tumors and the remodeling of the tumor microenvironment (Skeate et al., 2020). He et al. (2018) found TNFSF14–CGKRK could induce high endothelial venules formation and lymphocyte accumulation in murine glioblastoma (He et al., 2018). Brunetti et al. (2020) found that the expression of TNFSF14 in serum was higher in patients with bone metastases than in controls (Brunetti et al., 2020). TNFSF14 could promote osteolytic bone metastases in non-small cell lung cancer patients (Brunetti et al., 2020). In the present study, we found the expression of TNFSF14 was increased significantly in patients with KIRC and patients with KIRC with high-risk scores. Patients with KIRC with high expression of TNFSF14 exhibited worse OS. Our present studies further reinforce the relationship of TNFSF14 with cancer, immune characteristic, and survival status.

Although the risk model constructed by using these two signatures (SLC11A1 and TNFSF14) could better predict the prognosis of patients with KIRC, there are still some limitations in our present study, such as a small sample size and a lack of cross-validation. However, since we did not find other suitable data information of KIRC, we will collect a large number of clinical samples of KIRC to confirm the model. These will be our next focus of investigation.

## Conclusion

Epigenetic dysregulations are clearly associated with the development of renal cancer. In the present study, we not only identified two IR-DEGs may be the prognosis signatures for KIRC, but also provided a strategy for the screening of suitable prognosis signatures correlated with aberrant methylations for other cancers, even though the results require further validation.

## Data Availability

The original contributions presented in the study are included in the article/Supplementary Material, further inquiries can be directed to the corresponding author.
