# Supplementary material for: Identification of Two Immune Related Genes Correlated With Aberrant Methylations as Prognosis Signatures for Renal Clear Cell Carcinoma
Source: Front Genet. 2021 Dec 2;12:750997. doi: 10.3389/fgene.2021.750997 (PMC8674690; doi:10.3389/fgene.2021.750997)
Supplement: Supplementary file 6 [file DataSheet2.DOCX]

**Identification of two** **immune related genes correlated with aberrant methylations as prognosis signatures for renal clear cell carcinoma**

Zhi-Yong Yao^1,2#^, Chaoqung Xing^1#^, Yuan-Wu Liu^3^, Xiao-Liang Xing^1,2^*

^1^The first affiliated Hospital of Hunan University of Medicine, Huaihua 418000, Hunan, P. R. China.

^2^School of public health and laboratory medicine, Hunan University of Medicine, Huaihua 418000, Hunan, P. R. China.

^3^Beijing Advanced Innovation Center for Food Nutrition and Human Health, China Agricultural University, 100193, Beijing, China.

^#^contributed equally to this work.

*correspondence: Xiao-Liang Xing, xiaoliangxinghnm@126.com

**Supplementary information:** 1 figures and 3 tables.


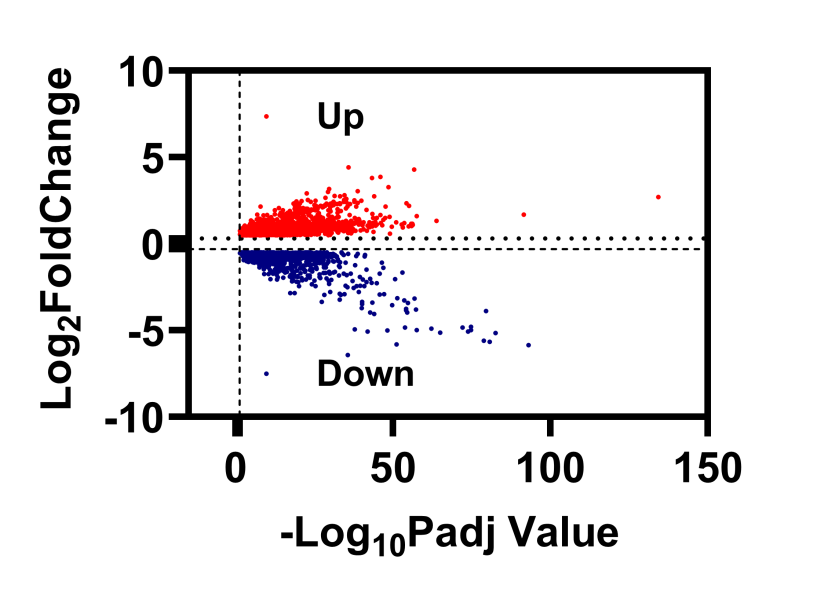


**Supplementary figure 1** Volcano plot of DEGs between patients with high risk value and patients with low risk value.

**Supplementary table 1** GO analyses for DEGs between normal and kidney cancer.

| **Term** | | **Count** | **GenesRatio** | **PValue** | **FDR** |
| --- | --- | --- | --- | --- | --- |
| **BP** | immune response | 180 | 6.27 | 0.0000 | 0.0000 |
|  | regulation of immune response | 99 | 3.45 | 0.0000 | 0.0000 |
|  | complement activation, classical pathway | 62 | 2.16 | 0.0000 | 0.0000 |
|  | inflammatory response | 133 | 4.64 | 0.0000 | 0.0000 |
|  | complement activation | 55 | 1.92 | 0.0000 | 0.0000 |
|  | Fc-gamma receptor signaling pathway involved in phagocytosis | 64 | 2.23 | 0.0000 | 0.0000 |
|  | receptor-mediated endocytosis | 77 | 2.68 | 0.0000 | 0.0000 |
|  | signal transduction | 248 | 8.64 | 0.0000 | 0.0000 |
|  | T cell costimulation | 39 | 1.36 | 0.0000 | 0.0000 |
|  | proteolysis | 129 | 4.50 | 0.0000 | 0.0000 |
|  | B cell receptor signaling pathway | 31 | 1.08 | 0.0000 | 0.0000 |
|  | innate immune response | 114 | 3.97 | 0.0000 | 0.0000 |
|  | chemotaxis | 48 | 1.67 | 0.0000 | 0.0000 |
|  | cell adhesion | 116 | 4.04 | 0.0000 | 0.0000 |
|  | cell surface receptor signaling pathway | 79 | 2.75 | 0.0000 | 0.0000 |
|  | positive regulation of T cell proliferation | 30 | 1.05 | 0.0000 | 0.0000 |
|  | adaptive immune response | 51 | 1.78 | 0.0000 | 0.0000 |
|  | transmembrane receptor protein tyrosine kinase signaling pathway | 38 | 1.32 | 0.0000 | 0.0000 |
|  | phagocytosis, engulfment | 21 | 0.73 | 0.0000 | 0.0000 |
|  | T cell activation | 24 | 0.84 | 0.0000 | 0.0000 |
|  | phagocytosis, recognition | 18 | 0.63 | 0.0000 | 0.0000 |
|  | chemokine-mediated signaling pathway | 30 | 1.05 | 0.0000 | 0.0000 |
|  | leukocyte migration | 42 | 1.46 | 0.0000 | 0.0000 |
|  | positive regulation of B cell activation | 17 | 0.59 | 0.0000 | 0.0000 |
|  | positive regulation of neutrophil chemotaxis | 15 | 0.52 | 0.0000 | 0.0000 |
|  | response to lipopolysaccharide | 49 | 1.71 | 0.0000 | 0.0000 |
|  | positive regulation of ERK1 and ERK2 cascade | 51 | 1.78 | 0.0000 | 0.0000 |
|  | Fc-epsilon receptor signaling pathway | 51 | 1.78 | 0.0000 | 0.0000 |
|  | positive regulation of cytosolic calcium ion concentration | 41 | 1.43 | 0.0000 | 0.0001 |
|  | cell chemotaxis | 25 | 0.87 | 0.0000 | 0.0004 |
|  | neutrophil chemotaxis | 25 | 0.87 | 0.0000 | 0.0005 |
|  | cellular defense response | 24 | 0.84 | 0.0000 | 0.0005 |
|  | positive regulation of interferon-gamma production | 20 | 0.70 | 0.0000 | 0.0005 |
|  | interferon-gamma-mediated signaling pathway | 26 | 0.91 | 0.0000 | 0.0005 |
|  | cell-cell signaling | 62 | 2.16 | 0.0000 | 0.0007 |
|  | cellular response to tumor necrosis factor | 34 | 1.19 | 0.0000 | 0.0009 |
|  | positive regulation of angiogenesis | 35 | 1.22 | 0.0000 | 0.0009 |
|  | integrin-mediated signaling pathway | 31 | 1.08 | 0.0000 | 0.0017 |
|  | extracellular matrix organization | 50 | 1.74 | 0.0000 | 0.0017 |
|  | defense response to bacterium | 40 | 1.39 | 0.0000 | 0.0021 |
|  | antigen processing and presentation of peptide or polysaccharide antigen via MHC class II | 11 | 0.38 | 0.0000 | 0.0022 |
|  | acute-phase response | 17 | 0.59 | 0.0000 | 0.0028 |
|  | positive regulation of NF-kappaB import into nucleus | 12 | 0.42 | 0.0000 | 0.0034 |
|  | positive regulation of NF-kappaB transcription factor activity | 37 | 1.29 | 0.0000 | 0.0035 |
|  | positive regulation of phagocytosis | 14 | 0.49 | 0.0000 | 0.0050 |
|  | platelet activation | 33 | 1.15 | 0.0000 | 0.0050 |
|  | negative regulation of T cell proliferation | 16 | 0.56 | 0.0000 | 0.0054 |
|  | T cell receptor signaling pathway | 39 | 1.36 | 0.0001 | 0.0069 |
|  | cellular response to lipopolysaccharide | 32 | 1.12 | 0.0001 | 0.0082 |
|  | platelet degranulation | 30 | 1.05 | 0.0001 | 0.0082 |
|  | positive regulation of phosphatidylinositol 3-kinase signaling | 22 | 0.77 | 0.0001 | 0.0091 |
|  | cytokine-mediated signaling pathway | 35 | 1.22 | 0.0001 | 0.0120 |
|  | regulation of cell proliferation | 45 | 1.57 | 0.0001 | 0.0121 |
|  | apoptotic process | 109 | 3.80 | 0.0001 | 0.0138 |
|  | ion transmembrane transport | 49 | 1.71 | 0.0002 | 0.0165 |
|  | excretion | 15 | 0.52 | 0.0002 | 0.0194 |
|  | ureteric bud development | 15 | 0.52 | 0.0003 | 0.0264 |
|  | T cell differentiation | 13 | 0.45 | 0.0003 | 0.0291 |
|  | cellular response to interleukin-1 | 22 | 0.77 | 0.0003 | 0.0316 |
|  | positive regulation of interleukin-2 biosynthetic process | 8 | 0.28 | 0.0004 | 0.0321 |
|  | negative regulation of immune response | 8 | 0.28 | 0.0004 | 0.0321 |
|  | negative regulation of growth | 10 | 0.35 | 0.0004 | 0.0343 |
|  | defense response to protozoan | 10 | 0.35 | 0.0004 | 0.0343 |
|  | antigen processing and presentation of exogenous peptide antigen via MHC class II | 26 | 0.91 | 0.0004 | 0.0356 |
|  | positive regulation of cell migration | 43 | 1.50 | 0.0004 | 0.0356 |
|  | positive regulation of interleukin-10 production | 11 | 0.38 | 0.0004 | 0.0367 |
|  | positive regulation of release of sequestered calcium ion into cytosol | 12 | 0.42 | 0.0005 | 0.0371 |
|  | positive regulation of inflammatory response | 22 | 0.77 | 0.0005 | 0.0415 |
|  | embryonic digestive tract development | 9 | 0.31 | 0.0005 | 0.0423 |
|  | lipopolysaccharide-mediated signaling pathway | 13 | 0.45 | 0.0006 | 0.0480 |
|  | positive regulation of interleukin-4 production | 10 | 0.35 | 0.0006 | 0.0480 |
|  | positive regulation of calcium-mediated signaling | 10 | 0.35 | 0.0006 | 0.0480 |
|  | negative regulation of endothelial cell apoptotic process | 12 | 0.42 | 0.0007 | 0.0492 |
| **CC** | plasma membrane | 822 | 28.65 | 0.0000 | 0.0000 |
|  | external side of plasma membrane | 101 | 3.52 | 0.0000 | 0.0000 |
|  | integral component of plasma membrane | 339 | 11.82 | 0.0000 | 0.0000 |
|  | extracellular region | 365 | 12.72 | 0.0000 | 0.0000 |
|  | extracellular space | 301 | 10.49 | 0.0000 | 0.0000 |
|  | integral component of membrane | 859 | 29.94 | 0.0000 | 0.0000 |
|  | blood microparticle | 51 | 1.78 | 0.0000 | 0.0000 |
|  | extracellular exosome | 474 | 16.52 | 0.0000 | 0.0000 |
|  | cell surface | 119 | 4.15 | 0.0000 | 0.0000 |
|  | apical plasma membrane | 72 | 2.51 | 0.0000 | 0.0000 |
|  | basolateral plasma membrane | 50 | 1.74 | 0.0000 | 0.0000 |
|  | collagen trimer | 31 | 1.08 | 0.0000 | 0.0001 |
|  | immunological synapse | 17 | 0.59 | 0.0000 | 0.0001 |
|  | proteinaceous extracellular matrix | 65 | 2.27 | 0.0000 | 0.0001 |
|  | immunoglobulin complex, circulating | 12 | 0.42 | 0.0000 | 0.0003 |
|  | platelet dense granule lumen | 10 | 0.35 | 0.0000 | 0.0007 |
|  | basement membrane | 26 | 0.91 | 0.0000 | 0.0009 |
|  | T cell receptor complex | 11 | 0.38 | 0.0000 | 0.0012 |
|  | MHC class II protein complex | 12 | 0.42 | 0.0000 | 0.0016 |
|  | membrane raft | 47 | 1.64 | 0.0003 | 0.0106 |
|  | transport vesicle membrane | 14 | 0.49 | 0.0009 | 0.0310 |
|  | membrane-bounded vesicle | 8 | 0.28 | 0.0011 | 0.0366 |
|  | receptor complex | 31 | 1.08 | 0.0012 | 0.0383 |
|  | alpha-beta T cell receptor complex | 5 | 0.17 | 0.0014 | 0.0432 |
| **MF** | antigen binding | 59 | 2.06 | 0.0000 | 0.0000 |
|  | serine-type endopeptidase activity | 95 | 3.31 | 0.0000 | 0.0000 |
|  | receptor activity | 62 | 2.16 | 0.0000 | 0.0000 |
|  | immunoglobulin receptor binding | 17 | 0.59 | 0.0000 | 0.0000 |
|  | cell adhesion molecule binding | 27 | 0.94 | 0.0000 | 0.0000 |
|  | receptor binding | 83 | 2.89 | 0.0000 | 0.0001 |
|  | chemokine activity | 22 | 0.77 | 0.0000 | 0.0001 |
|  | MHC class II receptor activity | 11 | 0.38 | 0.0000 | 0.0006 |
|  | carbohydrate binding | 51 | 1.78 | 0.0000 | 0.0006 |
|  | IgG binding | 9 | 0.31 | 0.0000 | 0.0020 |
|  | heparin binding | 42 | 1.46 | 0.0000 | 0.0031 |
|  | calcium ion binding | 135 | 4.71 | 0.0000 | 0.0031 |
|  | transmembrane signaling receptor activity | 50 | 1.74 | 0.0001 | 0.0112 |
|  | sodium channel activity | 9 | 0.31 | 0.0003 | 0.0333 |
|  | phosphatidylinositol-4,5-bisphosphate 3-kinase activity | 20 | 0.70 | 0.0003 | 0.0357 |
|  | monooxygenase activity | 19 | 0.66 | 0.0004 | 0.0392 |
|  | phosphatidylinositol phospholipase C activity | 12 | 0.42 | 0.0004 | 0.0392 |
|  | cytokine activity | 41 | 1.43 | 0.0004 | 0.0392 |
|  | Ras guanyl-nucleotide exchange factor activity | 30 | 1.05 | 0.0004 | 0.0392 |
|  | calmodulin binding | 43 | 1.50 | 0.0005 | 0.0441 |

**Supplementary table 2** KEGG analyses for DEGs between normal and kidney cancer.

| **Term** | **Count** | **GenesRatio** | **PValue** | **FDR** |
| --- | --- | --- | --- | --- |
| Staphylococcus aureus infection | 39 | 1.36 | 0.0000 | 0.0000 |
| Cytokine-cytokine receptor interaction | 86 | 3.00 | 0.0000 | 0.0000 |
| Rheumatoid arthritis | 45 | 1.57 | 0.0000 | 0.0000 |
| Cell adhesion molecules (CAMs) | 58 | 2.02 | 0.0000 | 0.0000 |
| Phagosome | 58 | 2.02 | 0.0000 | 0.0000 |
| Hematopoietic cell lineage | 38 | 1.32 | 0.0000 | 0.0000 |
| Complement and coagulation cascades | 33 | 1.15 | 0.0000 | 0.0000 |
| Graft-versus-host disease | 21 | 0.73 | 0.0000 | 0.0000 |
| Type I diabetes mellitus | 23 | 0.80 | 0.0000 | 0.0000 |
| Leishmaniasis | 31 | 1.08 | 0.0000 | 0.0000 |
| Allograft rejection | 21 | 0.73 | 0.0000 | 0.0000 |
| Inflammatory bowel disease (IBD) | 28 | 0.98 | 0.0000 | 0.0000 |
| Osteoclast differentiation | 44 | 1.53 | 0.0000 | 0.0000 |
| Tuberculosis | 52 | 1.81 | 0.0000 | 0.0000 |
| Primary immunodeficiency | 18 | 0.63 | 0.0000 | 0.0000 |
| Intestinal immune network for IgA production | 21 | 0.73 | 0.0000 | 0.0000 |
| NF-kappa B signaling pathway | 30 | 1.05 | 0.0000 | 0.0001 |
| Viral myocarditis | 22 | 0.77 | 0.0000 | 0.0004 |
| Malaria | 20 | 0.70 | 0.0000 | 0.0004 |
| Chemokine signaling pathway | 49 | 1.71 | 0.0000 | 0.0005 |
| Chagas disease (American trypanosomiasis) | 32 | 1.12 | 0.0001 | 0.0006 |
| Autoimmune thyroid disease | 20 | 0.70 | 0.0001 | 0.0008 |
| Amoebiasis | 32 | 1.12 | 0.0001 | 0.0008 |
| Fc gamma R-mediated phagocytosis | 27 | 0.94 | 0.0001 | 0.0010 |
| Pertussis | 25 | 0.87 | 0.0001 | 0.0010 |
| Asthma | 14 | 0.49 | 0.0001 | 0.0013 |
| T cell receptor signaling pathway | 30 | 1.05 | 0.0002 | 0.0014 |
| Legionellosis | 19 | 0.66 | 0.0004 | 0.0037 |
| Aldosterone-regulated sodium reabsorption | 15 | 0.52 | 0.0008 | 0.0067 |
| Calcium signaling pathway | 43 | 1.50 | 0.0010 | 0.0081 |
| Pathways in cancer | 80 | 2.79 | 0.0015 | 0.0117 |
| Toxoplasmosis | 29 | 1.01 | 0.0019 | 0.0143 |
| Antigen processing and presentation | 22 | 0.77 | 0.0023 | 0.0168 |
| HTLV-I infection | 55 | 1.92 | 0.0024 | 0.0168 |
| Neuroactive ligand-receptor interaction | 59 | 2.06 | 0.0025 | 0.0168 |
| Systemic lupus erythematosus | 33 | 1.15 | 0.0029 | 0.0192 |
| Mineral absorption | 15 | 0.52 | 0.0030 | 0.0195 |
| Collecting duct acid secretion | 11 | 0.38 | 0.0034 | 0.0216 |
| Natural killer cell mediated cytotoxicity | 30 | 1.05 | 0.0047 | 0.0292 |
| NOD-like receptor signaling pathway | 17 | 0.59 | 0.0052 | 0.0314 |
| Leukocyte transendothelial migration | 28 | 0.98 | 0.0075 | 0.0439 |

**Supplementary table 3** Differentially expressed immune cells and molecules between normal and KIRC cancer

| **Type_cell** | | **Normal (n=72)** | | **Cancer (n=530)** | |
| --- | --- | --- | --- | --- | --- |
|  |  | **Mean** | **STD** | **Mean** | **STD** |
| CIBERSORT | B cell naive | 1.00 | 0.62 | 0.15 | 0.25 |
|  | T cell CD4+ memory resting | 1.00 | 0.27 | 0.57 | 0.35 |
|  | Macrophage M1 | 1.00 | 0.75 | 2.35 | 1.28 |
|  | T cell CD8+ | 1.00 | 0.71 | 3.34 | 2.32 |
|  | T cell follicular helper | 1.00 | 2.09 | 5.07 | 4.74 |
|  | Mast cell activated | 1.00 | 0.96 | 0.47 | 0.63 |
|  | B cell plasma | 1.00 | 0.71 | 0.61 | 0.47 |
|  | Monocyte | 1.00 | 0.65 | 0.62 | 0.61 |
|  | Macrophage M2 | 1.00 | 0.37 | 1.25 | 0.44 |
|  | T cell gamma delta | 1.00 | 2.11 | 4.55 | 7.30 |
|  | T cell regulatory (Tregs) | 1.00 | 2.16 | 2.15 | 2.79 |
|  | NK cell resting | 1.00 | 3.40 | 4.84 | 11.60 |
|  | Macrophage M0 | 1.00 | 5.17 | 13.61 | 44.15 |
|  | Eosinophil | 1.00 | 4.05 | 0.27 | 2.27 |
|  | NK cell activated | 1.00 | 0.63 | 0.85 | 0.53 |
|  | Mast cell resting | 1.00 | 1.89 | 0.62 | 1.40 |
| CIBERSORT-ABS | B cell naive | 1.00 | 0.69 | 0.33 | 0.49 |
|  | Macrophage M2 | 1.00 | 0.81 | 2.48 | 1.32 |
|  | Macrophage M1 | 1.00 | 0.92 | 5.16 | 3.86 |
|  | T cell CD8+ | 1.00 | 0.96 | 7.03 | 6.57 |
|  | T cell follicular helper | 1.00 | 2.19 | 13.18 | 13.93 |
|  | NK cell activated | 1.00 | 0.63 | 2.17 | 1.69 |
|  | T cell regulatory (Tregs) | 1.00 | 1.93 | 6.63 | 8.73 |
|  | T cell gamma delta | 1.00 | 2.55 | 8.91 | 15.62 |
|  | B cell plasma | 1.00 | 0.72 | 1.55 | 1.37 |
|  | Macrophage M0 | 1.00 | 4.99 | 28.68 | 82.82 |
|  | NK cell resting | 1.00 | 4.15 | 5.97 | 14.87 |
| EPIC | T cell CD4+ | 1.00 | 0.17 | 0.43 | 0.27 |
|  | Endothelial cell | 1.00 | 0.79 | 2.85 | 1.89 |
|  | Macrophage | 1.00 | 0.82 | 6.14 | 5.87 |
|  | uncharacterized cell | 1.00 | 0.07 | 0.86 | 0.17 |
|  | NK cell | 1.00 | 8.48 | 30.63 | 65.82 |
|  | Cancer associated fibroblast | 1.00 | 0.95 | 2.52 | 3.98 |
| MCPCOUNTER | T cell | 1.00 | 0.54 | 0.17 | 0.18 |
|  | Monocyte | 1.00 | 0.60 | 2.65 | 1.19 |
|  | Macrophage/Monocyte | 1.00 | 0.60 | 2.65 | 1.19 |
|  | NK cell | 1.00 | 0.97 | 3.72 | 2.07 |
|  | Neutrophil | 1.00 | 0.34 | 0.64 | 0.28 |
|  | cytotoxicity score | 1.00 | 1.19 | 8.11 | 6.49 |
|  | Endothelial cell | 1.00 | 0.42 | 2.11 | 1.25 |
|  | T cell CD8+ | 1.00 | 1.04 | 9.41 | 12.50 |
|  | Myeloid dendritic cell | 1.00 | 0.74 | 1.53 | 0.90 |
| QUANTISEQ | Macrophage M1 | 1.00 | 1.24 | 5.51 | 3.59 |
|  | T cell CD4+ (non-regulatory) | 1.00 | 2.05 | 5.17 | 4.11 |
|  | Neutrophil | 1.00 | 0.48 | 0.72 | 0.29 |
|  | T cell CD8+ | 1.00 | 2.52 | 57.44 | 87.05 |
|  | Macrophage M2 | 1.00 | 0.30 | 1.38 | 0.61 |
|  | uncharacterized cell | 1.00 | 0.08 | 0.93 | 0.12 |
|  | T cell regulatory (Tregs) | 1.00 | 0.71 | 0.79 | 0.74 |
|  | Myeloid dendritic cell | 1.00 | 1.25 | 0.75 | 0.97 |
|  | NK cell | 1.00 | 0.30 | 0.89 | 0.45 |
| TIMER | Neutrophil | 1.00 | 1.60 | 4.86 | 3.45 |
|  | Myeloid dendritic cell | 1.00 | 0.44 | 1.79 | 0.79 |
|  | T cell CD8+ | 1.00 | 1.31 | 6.03 | 5.28 |
|  | T cell CD4+ | 1.00 | 0.51 | 1.41 | 0.85 |
|  | Macrophage | 1.00 | 1.79 | 2.23 | 3.15 |
|  | B cell | 1.00 | 0.65 | 1.34 | 0.96 |
| XCELL | T cell NK | 1.00 | 2.58 | 18.66 | 9.28 |
|  | T cell CD4+ effector memory | 1.00 | 5.75 | 31.64 | 17.94 |
|  | Myeloid dendritic cell activated | 1.00 | 1.13 | 3.01 | 1.23 |
|  | Hematopoietic stem cell | 1.00 | 0.31 | 0.51 | 0.31 |
|  | microenvironment score | 1.00 | 0.54 | 2.56 | 1.11 |
|  | T cell CD4+ Th1 | 1.00 | 0.29 | 1.94 | 0.80 |
|  | Endothelial cell | 1.00 | 0.47 | 2.25 | 1.14 |
|  | Monocyte | 1.00 | 4.10 | 18.51 | 16.15 |
|  | immune score | 1.00 | 2.16 | 19.40 | 17.33 |
|  | Mast cell | 1.00 | 3.03 | 12.14 | 12.40 |
|  | T cell CD8+ naive | 1.00 | 8.49 | 312.03 | 361.02 |
|  | T cell CD8+ central memory | 1.00 | 4.71 | 47.39 | 56.81 |
|  | Macrophage | 1.00 | 1.93 | 9.30 | 10.38 |
|  | T cell CD8+ | 1.00 | 8.49 | 915.36 | 1193.71 |
|  | T cell CD8+ effector memory | 1.00 | 8.49 | 78.69 | 104.51 |
|  | Macrophage M1 | 1.00 | 4.25 | 25.29 | 35.68 |
|  | Macrophage M2 | 1.00 | 0.55 | 1.73 | 1.13 |
|  | Myeloid dendritic cell | 1.00 | 2.48 | 3.55 | 4.34 |
|  | stroma score | 1.00 | 0.51 | 1.41 | 0.72 |
|  | B cell plasma | 1.00 | 2.76 | 10.45 | 17.12 |
|  | Class-switched memory B cell | 1.00 | 1.84 | 2.08E+15 | 3.80E+15 |
|  | B cell | 1.00 | 8.49 | 651.43 | 1267.11 |
|  | Plasmacytoid dendritic cell | 1.00 | 8.30 | 165.20 | 321.28 |
|  | T cell CD4+ central memory | 1.00 | 1.71 | 2.36E+15 | 4.69E+15 |
|  | T cell CD4+ Th2 | 1.00 | 5.59 | 8.82 | 18.63 |
|  | Cancer associated fibroblast | 1.00 | 1.65 | 0.46 | 1.20 |
|  | T cell CD4+ naive | 1.00 | 1.81 | 1.27E+15 | 3.61E+15 |
|  | B cell naive | 1.00 | 1.70 | 5.15E+14 | 1.51E+15 |
|  | T cell CD4+ memory | 1.00 | 6.01 | 21.45 | 74.40 |
|  | Common lymphoid progenitor | 1.00 | 0.44 | 0.87 | 0.48 |
|  | Eosinophil | 1.00 | 1.71 | 2.07E+14 | 8.16E+14 |
